# P03 Not everything is lupus

**DOI:** 10.1093/rap/rkab068.002

**Published:** 2021-10-19

**Authors:** Jonathan Pinnell, Sofia Tosounidou

**Affiliations:** Sandwell and West Birmingham NHS Trust, Birmingham, United Kingdom

## Abstract

**Case report - Introduction:**

The association between malignancy and rheumatic diseases is well established.  Systemic lupus erythematosus (SLE) is no exception and is known to have associations with lymphoma and solid cancers of the lung, liver, and thyroid.  Non-Hodgkin’s lymphoma is the commonest malignancy with a standardised incidence ratio of 4.4—5.7.  However, they can present atypically, at any time, and can easily be confused with flares of SLE, so the rheumatologist must remain vigilant.

We present a case of Sezary Syndrome masquerading as cutaneous lupus that presented 23 years after the initial diagnosis of SLE.

**Case report - Case description:**

A 57-year-old black female had anti-nuclear antigen and anti-Ro antibody positive SLE.  In 2017, she attended with arthritis and a persistent severe malar and widespread lupus rash affecting her arms, chest, abdomen, and legs.  She was taking mycophenolate, hydroxychloroquine, and prednisolone.  She had previously experienced recurrent arthritis, oral ulcers, skin lesions, secondary Sjogren's Syndrome, episcleritis, leukopenia and Raynaud's phenomenon, and had found methotrexate and ciclosporin ineffective.  However, epratuzumab (anti-CD22 antibody), given during the EMBODY trial, had helped her symptoms. She was therefore given rituximab, which helped her arthritis but her cutaneous features got worse.

In March 2018 her dermatologist described a widespread erythema with adherent scales covering her back, abdomen and legs that had been preceded by vesicles and pustules on her chest, abdomen and thighs.  She was given emollients and topical corticosteroids.  A biopsy from her lower back showed abnormal and atypical CD4 positive T cells with cerebriform nuclei in the epidermis, follicular epithelium, and superficial dermis, with increased papillary dermal fibrosis. These features were felt to be in keeping with patch stage Mycosis fungoides.  She was referred to the skin lymphoma clinic where a scalp biopsy showed features of cutaneous lupus but a later thigh biopsy confirmed that she also had Mycosis fungoides. A staging CT confirmed axillary lymphadenopathy and blood tests showed Sezary cells, suggesting Sezary Syndrome.

As lupus prevented the use of UVB light therapy, she was given methotrexate but her skin rash progressed to Stage IV Mycosis Fungoides. She developed side effects with gemcitabine so moved to a cyclophosphamide, doxorubicin, vincristine and prednisolone chemotherapy regimen which helped her skin and SLE. However, her skin progressed again when this stopped. She had shown only a partial response to mogamulizumab (anti-CCR4 antibody) and so she is currently being considered for extracorporeal photopheresis as a last treatment option.

**Case report - Discussion:**

A prolonged history of SLE with arthritis and mucocutaneous features made the diagnosis of Sezary Syndrome more complex in this case and may have delayed the diagnosis. However, the diagnosis was astutely reconsidered when rituximab failed to induce skin improvement. This prompted appropriate referrals to the dermatologist and thereafter for a skin biopsy. Coexisting cutaneous lupus on the scalp biopsy further complicated the diagnosis but strong clinical suspicion led to the confirmation of the diagnosis of Sezary Syndrome on further skin biopsy and blood tests.

Mycosis fungoides is rare but it represents 60—70% of all cutaneous T-cell lymphomas. It typically presents in patients aged 50—60 with a chronic itch and fine scaling erythematous patches that can spread and thicken. Black patients can present at a younger age, they may have hypopigmented lesions, and the lesions may be more widespread and aggressive. Patients may have ‘B symptoms’ as occur in other lymphomas. The diagnosis is often delayed, requiring multiple biopsies.  Biopsies show cutaneous infiltration of malignant CD4 T-cells. The condition contrasts with cutaneous lupus as it typically affects sun-protected sites.

Sezary Syndrome can appear similar to Mycosis fungoides but typically presents with erythroderma and lymphadenopathy. Histology is also similar but can show acanthosis, dermal fibrosis, and cerebriform nuclear atypia as in this case.  Sezary cells have a cerebriform nucleus and can be detected in blood or on lymph node biopsy.  Unlike Mycosis fungoides which is an indolent condition with a 5-year survival of 87%, Sezary Syndrome is more aggressive with a 5-year survival less than 30%.  It is therefore important that cutaneous lymphoma be considered in any patient with autoimmune disease who has cutaneous features that behave atypically.

**Case report - Key learning points:**

Patients with rheumatic diseases have an increased risk of developing malignancy. These may occur at any time and may be obscured by flares of the rheumatic disease.  As in this case, features may be similar to the rheumatic disease but rheumatologists must be vigilant and responsive to atypical patterns or atypical responses to treatment. Other specialties may also need support when investigating such patients as atypical features may be less apparent if they are unfamiliar with the rheumatic condition leading to symptoms being attributed to the rheumatic disease. As in this case, seek confirmation of the diagnosis, ideally with tissue histology, when the diagnosis remains uncertain.

Mycosis fungoides and Sezary Syndrome are rare but are thought to be associated with chronic antigen stimulation so may be more common in autoimmune disease, especially SLE. We highlight these conditions so that they may be considered by the rheumatologist in the differential diagnosis of any patient presenting with an itchy, scaling, erythematous rash.

